# Interprofessional learning in social and health care—Learning experiences from large‐group simulation in Finland

**DOI:** 10.1002/nop2.589

**Published:** 2020-08-05

**Authors:** Terhi Saaranen, Marja Silén‐Lipponen, Maria Palkolahti, Kaarina Mönkkönen, Miia Tiihonen, Marjorita Sormunen

**Affiliations:** ^1^ Department of Nursing Science University of Eastern Finland Kuopio Finland; ^2^ School of Health Care Savonia University of Applied Sciences Kuopio Finland; ^3^ Faculty of Social Sciences University of Eastern Finland Kuopio Finland; ^4^ School of Pharmacy University of Eastern Finland Kuopio Finland; ^5^ School of Medicine Public Health and Clinical Nutrition Institute of Public Health University of Eastern Finland Kuopio Finland

**Keywords:** interprofessional collaboration, learning, nurses, nursing simulation, teaching

## Abstract

**Aim:**

This study aimed to describe the learning experiences of social and healthcare students and professionals of an interprofessional large‐group simulation. A simulation on sudden infant death syndrome (SIDS) was organized in collaboration between a Finnish university, university hospital and university of applied sciences.

**Design:**

A case study.

**Methods:**

The research data were collected at the large‐group simulation with a questionnaire containing variables on a five‐point Likert scale and open questions. The questionnaire was filled out by 350 students and professionals participating in the simulation. The quantitative data were analysed using descriptive statistical methods and the open‐ended questions by inductive content analysis.

**Results:**

The large‐group simulation proved to be a valid teaching and learning method for collaborating with other professionals and interacting with clients and the method can be considered as cost‐effective compared with small‐group simulations. The produced knowledge can be used in planning simulations in basic and in‐service training.

## 1 INTRODUCTION

The health problems of clients and patients are becoming more and more complex. The health sector is undergoing continuous changes, involving increasingly diverse health environments and situations. As a result, there is an increasing need for multiprofessional cooperation, communication and the combination of knowledge and expertise. This enables meeting today's challenges through the special competence of different professionals and producing good and safe care for clients and patients (Labrague, McEnroe‐Petitte, Fronda, & Obeidat, 2018; McCave, Aptaker, Hartman, & Zucconi, 2019). Good collaboration between professional groups requires, among other things, knowledge of the competence of other professions as well as communication to ensure that all professional groups understand the common goal of their work and each other's’ working approaches (Ponzer & Castrén, 2013). The Interprofessional Education Collaborative (IPEC, 2016) has defined the core areas of expertise for interprofessional collaboration, including values and ethics, roles and responsibilities, communication and teamwork. To develop the expertise of interprofessional collaboration, the role of multidisciplinary learning and teaching between different professions is systematically highlighted (Cahill, O’Donell, Warren, Taylor, & Gowan, 2013; World Health Organization (WHO), 2010). However, the education in social and health care rarely provides opportunities for practicing and developing interprofessional collaboration skills before students transition to the working life, as the training of professionals mainly takes place in their specific professional fields (Costello et al., 2018) and mostly focuses on the technical and substantive aspects of work. This can cause difficulties in working in interprofessional teams after graduation if newly graduated professionals have no previous experience of such collaboration as they enter the workforce (Johnston, Coyer, & Nash, 2017; Palese et al., 2019). This makes it important to include more training on interprofessional cooperation in various study programs.

## 2 BACKGROUND

Interprofessional teaching promotes opportunities for learning approaches that enable different professional groups to work together in an aim to address clients’ care needs (Centre of Advancement of Interprofessional Education (CAIPE), 2017). Interprofessional teaching can be implemented in many different ways, for example, through joint lectures, group work, clinical practice (Homeyer, Hoffmann, Hingst, Oppermann, & Dreier‐Wolfgramm, 2018; Palese et al., 2019) and simulations (Pinto, Possanza, & Karpa, 2018). Simulation, both in clinical and university settings, provides authentic learning possibilities through, for instance, realistic simulation scenarios using genuine patient cases and current status descriptions and standardized patients or simulators who mimic the reality of patient situations. (Labrague et al., 2018; Mai et al., 2019; Naismith, Kowalski, Soklaridis, Kelly, & Walsh, 2020). Moreover, simulations provide a safe context for practicing healthcare scenarios that enable imitating complex cases in healthcare environments nearly identically to their real‐life counterparts without causing any risk to real patients (Roberts & Goodhand, 2018).

Interprofessional simulations where professionals from several fields work together in a realistic setting (Mai et al., 2019) have been found to be effective in developing collaborative expertise. As a teaching method, simulation allows the participants to practice their training contents and helps them combine theory with practice (Aura, Jordan, Saano, Tossavainen, & Turunen, 2016). The simulation makes it possible to repeat actions and provide open feedback and supports joint reflection, which makes it a well‐suited method for developing both safety‐critical skills (Mai et al., 2019) as well as interaction and interprofessional teamwork skills (Liaw, Zhou, Lau, Siau, & Chan, 2014).

The experiences obtained from interprofessional simulations have been mostly positive (Costello et al., 2018; Ivey, Bowman, & Lockeman, 2018; Jakobsen et al., 2018; Popkess et al., 2017; Roberts & Goodhand, 2018; Zamjahn et al., 2018), and there have been few differences in the participants’ satisfaction levels based on different occupational groups or sectors. However, in a study by Jakobsen et al. (2018), medical students were found to learn more than nursing students about the leader's role during a simulation. By contrast, in Popkess et al. (2017), nursing students were more satisfied with a simulation as a learning method than dentistry and pharmacy students, even though the dentistry and pharmacy students were more knowledgeable than the nursing students in this study.

The participants in interprofessional simulations have appreciated the related opportunity for learning in situations that resemble reality (Costello et al., 2018; Hovland, Whitford, & Niederriter, 2018; Naismith et al., 2020) and allow applying theory to practice (Hovland et al., 2018). Professional simulation training has supported the learning of high‐quality care and the development of the learners’ professional growth (Roberts & Goodhand, 2018). The participants have found that the simulations have increased their confidence in their own skills (Hovland et al., 2018; Jakobsen et al., 2018; Smith et al., 2018) and helped them prioritize patient needs (Roberts & Goodhand, 2018; Smith et al., 2018). Simulations have also allowed the participants to increase their understanding of the roles and responsibilities of other professionals (Bradway et al., 2018; Costello et al., 2018; Pinto et al., 2018; Wietholter et al., 2017; Zamjahn et al., 2018). Furthermore, interprofessional simulations have developed the participants’ leadership skills (Jakobsen et al., 2018; Pinto et al., 2018) and improved their stress management ability (Jakobsen et al., 2018).

Although the experiences of interprofessional simulations have been mostly positive, the practice has at times also included negative experiences. In a study by Van Schaik, Plant, and O’Brien (2015), while specialized doctors considered training carried out as a recovery simulation with nurses as a good way of learning, this also caused them anxiety and uncertainty. They appreciated the interprofessional training, but questioned the related interprofessional learning discussion, as they found maintaining a friendly and safe atmosphere and giving constructive feedback challenging and a source of tension. In a study by Jakobsen et al. (2018), nursing and medical students described the simulation they had participated in as fun and inspiring, but also stressful and partly embarrassing.

Usually, simulations are carried out in small groups with a few participants (Carson & Harder, 2016) or in groups of approximately 20–30 people (see Carson & Harder, 2016; Rode, Callihan, & Barnes, 2016). A large group can also be divided into small groups which participate in exercises separately (see Norman, Thompson, & Missildine, 2013). One obstacle for more extensive use of simulations in interprofessional teaching is concerned with the time required for the exercise. In small groups, interprofessional simulations must be repeated to allow all students in the interprofessional group to access and participate in the exercises (Homeyer et al., 2018). In addition, educational institutions often require for the training premises to be booked and the working hours of both students and staff to be planned considerably early in advance, up to a year before the implementation of the simulation. A further problem is concerned with how the simulations could be integrated into the curriculum without reducing the resources of other learning tasks. The time constraints included in the teachers’ work can lead to the teachers sticking to such time‐consuming teaching methods, as a result reducing potential interprofessional learning opportunities (Homeyer et al., 2018).

Due to challenges related to resources (time, space and tools) in organizing a small group simulation, recent efforts have been made to develop large group simulations accommodating hundreds of participants. A large group simulation follows the pedagogical structure of a small group simulation (Bambini, 2016) and progresses from orientation to implementation and final debriefing. The difference between a large and small group simulations emerges in that a large group simulation uses professional actors hired for the task, standardized patients or/and professionals. The learners observing a large simulation, that is the audience may be students or professionals participating in in‐service training. They are given an opportunity to participate in the debriefing by commenting and asking and answering questions through solutions such as their smartphones or a similar browser‐based participation system (Silén‐Lipponen, Tiihonen, Kekoni, & Saaranen, 2019).

As large group simulations are still under development, very few studies have investigated the implementation of the teaching and learning in an interprofessional simulation for large groups with over 30 participants (Rochester et al., 2012; Rode et al., 2016). Moreover, there is no information available on how participants experience learning in interprofessional large‐group simulations. Developing the teaching and learning in interprofessional large‐group simulations requires evidence‐based knowledge of the experience of participants representing different professions as well as the importance of background information for learning in a large‐group simulation (Nyström, Dahlberg, Hult, & Abrandt Dahlgren, 2016).

In this study involving implementing a large simulation, sudden infant death syndrome (SIDS) was chosen as the topic of the interprofessional simulation scenario. The choice was based on the need to explore and learn about the actions of different professionals in an acute crisis (e.g. Endacott et al., 2015; Jakobsen et al., 2018) and particularly the essential skill of collaboration, for which a large‐group simulation provided a good platform. The purpose of this study was to describe the learning experiences of students and professionals participating in an interprofessional large‐group simulation. The research questions were as follows:

1. How were the students and professionals’ learning experiences related to their background and how did they perceive the interprofessional large‐group simulation?
2. Which factors were particularly considered to promote learning in a large‐group simulation?

## 3 METHODS

### 3.1 Design

The case study approach was used in an aim to understand a particular case and involved in‐depth and comprehensive analysis of a social unit (Polit & Beck, 2018) and carrying out an interprofessional large‐group simulation. The simulation was held in November 2017 in collaboration between a Finnish university, university hospital and university of applied sciences.

### 3.2 Participants and context

The simulation participants were students and professionals representing the rescue sector, the police force, parish workers and several services in the social and healthcare sector. The simulation was included in the students’ curricula, and professionals were also invited to participate in it. In total, 427 students and professionals enrolled in the event and participated as observers in the simulation. The all participants could take part in the simulation in a lecture hall, or at three distant locations, with instructors present. Before the event, the participants received material about the simulation arrangements as well as instructions about incorporating their own mobile devices with an internet connection in the teaching situation.

The SIDS scenario was designed and scripted for the large‐group simulation in a team consisting of members from the above organizations. The setting for the scenario was a home with two parents and their infant child sleeping in a crib. Professional actors played the parents and professionals from pre‐hospital care, the police force, social care and church performed their own roles. The simulation event was started with a brief introduction, after which the scenario began with one of the parents finding their baby lifeless in the crib. The scenario proceeded with various encounters between parents and professionals and between different professionals. After an approximately 30‐min simulation scenario, the instructor leading the simulation held a debriefing session. All the professionals participating in the scenario also took part in the debriefing, which had the duration of roughly 40 min. The participants who followed the scenario from the lecture hall or remotely were able to join the discussion anonymously in real‐time during the debriefing using a mobile application. Any comments made by the participants were displayed on a large screen in the lecture hall and were visible for everyone at the event. After the debriefing session, a university psychologist and hospital pastor discussed the emotions than can emerge during a crisis and how to cope with these.

### 3.3 Instrument and data collection

The questionnaire used in this study included 14 questions and was developed in an interdisciplinary group including university research and teaching personnel from the disciplines of pharmacy, nursing, medicine and social sciences. Earlier findings related to interprofessional education (e.g. Aase, Hansen, Aase, & Reeves, 2016; Buckley et al., 2012) were used as the basis of the development process. In addition to the eight background questions (gender, age, occupational status, stage of studies, work experience, previous participation in a simulation, previous participation in an interprofessional simulation, previous knowledge about simulation events/rehearsals), the questionnaire included one structured question with nine five‐point Likert‐scale variables (totally agree—totally disagree) about the experiences and issues learned during the large‐group simulation and five open‐ended questions concerning the participants’ views on the simulation event; specifically on their satisfaction, encountering the client, cooperation between the professionals, developmental issues and suggestions for future simulation contents. This article reports the results of the participants’ experiences through one structured question (satisfaction, suitability of content, knowledge, interest and usefulness) and one open‐ended question (overall satisfaction with the interprofessional simulation).

All of the participants (students and professionals) were given paper questionnaires after the debriefing. They were asked to fill out the questionnaires in the lecture hall or the remote locations. The instructors collected the completed questionnaires.

### 3.4 Data analysis

The analysis of the quantitative data focused on using descriptive statistics. The data were analysed using IBM Statistics version 24. The five‐point Likert scale variables were classified into three categories (agree, cannot say and disagree). The background variables were described using frequencies and percentages and variables related to the simulation experiences using percentages, means and standard deviations. The Mann–Whitney *U* test was used to find out the possible statistical significance or differences between the participants’ background variables and simulation experiences. A significance level of 0.05 was adopted for statistical analyses. Qualitative data were analysed by inductive content analysis, and the data were presented qualitatively according to the research task. Firstly, authentic expressions were simplified and grouped according to their content. Simplified expressions with similar content were grouped and classified into 11 subcategories which were named according to their content. The subcategories were then compared and categories with similar content were integrated into four upper categories, which were again integrated as one main category (Moule & Goodman, 2013; Polit & Beck, 2018).

### 3.5 Ethical considerations

Ethical consideration took place throughout the data collection and analysis processes. The research process was granted ethical permission (Statement 6/2016) by the Committee on Research Ethics of the University of Eastern Finland. Participation in the study was voluntary and the research data were collected, analysed and registered without personal identifying data (Finnish Advisory Board on Research Integrity, 2012). The data were stored in the protected cloud storage service of the University of Eastern Finland in accordance with the university's guidelines.

## 4 RESULTS

### 4.1 Participants’ background characteristics

Of a total of 427 participants, 350 persons responded to the questionnaire (82%). Most participants were students (68%), of whom 27.5% studied social work or social science (university) and 21.5% were nursing students (university of applied sciences) (Table 1). Of the participants, 68% were students, 23% were professionals and 9% were both professionals and students. The most common professional field (42%) involved nursing (e.g. registered or practical nurse, midwife).

**Table 1:** Participants’ background characteristics (n, %)

Characteristics	*N*	%
Gender (*n* = 348)		
Female	297	85
Male	51	15
Age group (years) (*n* = 350)		
‐30	192	55
31–40	86	24.5
41‐	72	20.5
Student or professional (*n* = 349)		
Student	236	68
Professional	81	23
Both student and professional	32	9
Study program (*n* = 265)		
Social work or social science (university)	73	27.5
Nursing (university of applied sciences)	57	21.5
Nursing science (university)	29	11
Medicine	24	9
Paramedic nursing	20	7.5
Midwife or public health nursing	19	7
Pharmacy	18	6.5
Social work (university of applied sciences)	8	3
Psychology	4	1.5
Theology	4	1.5
Practical licensed nursing	4	1.5
Emergency response centre operator	2	1
Social psychology	2	1
Health promotion	1	0.5
Professional field (*n* = 114)		
Nursing	48	42
Emergency response centre operation	13	11.5
Social work	13	11.5
Nursing management and teaching	11	10
Theology	10	9
Medicine	6	5
Pharmacy	4	3.5
Law enforcement	4	3.5
Some other professional field	5	4

### 4.2 Participants’ experiences and factors promoting learning in the large group simulation

The participants’ experiences of the large group simulation were measured with statements using Likert scales (Table 2) and one open‐ended question. Both professional and student participants had positive experiences of the large group simulation (Table 2). Almost all the participants (95%) were satisfied with the experience. Most (83%) found that the large group simulation had increased their knowledge of simulation learning and that they (85%) had also become more interested in simulation learning. Nearly, all the participants thought that they had the learning experience brought benefits to their work or studies. There were no statistical differences between the experiences of the professional and student participants (Table 2; *p*‐values).

**Table 2:** Participants’ experiences of the large group simulation (%, mean, Standard Deviation, *p*‐value)

Experience of the large group simulation	Agree%	Neither agree nor disagree%	Disagree%	Mean	*SD*	*p*‐value
I am satisfied with the large group simulation as a whole (*n* = 348)	95	2	2	4.57	0.686	.745
The contents of the large group simulation formed a successful whole (*n* = 348)	95	2	2	4.47	0.705	.457
My knowledge of simulation learning and teaching increased during the large group simulation (*n* = 350)	83	12	5	4.22	0.964	.633
My interest in simulation learning and teaching increased during the large group simulation (*n* = 350)	85	12	3	4.33	0.846	.742
I believe that the large group simulation was useful for my work or studies (*n* = 350)	95	3	2	4.56	0.714	.147

Note

Mean scale 1 = strongly disagree. 0.5 = strongly agree, *SD* = standard deviation. *p*‐value = Mann–Whitney test to compare differences in students’ and professionals’ experiences of the large group simulation. (*p*‐value < .05 = statistically significant).

**FIGURE 1:**
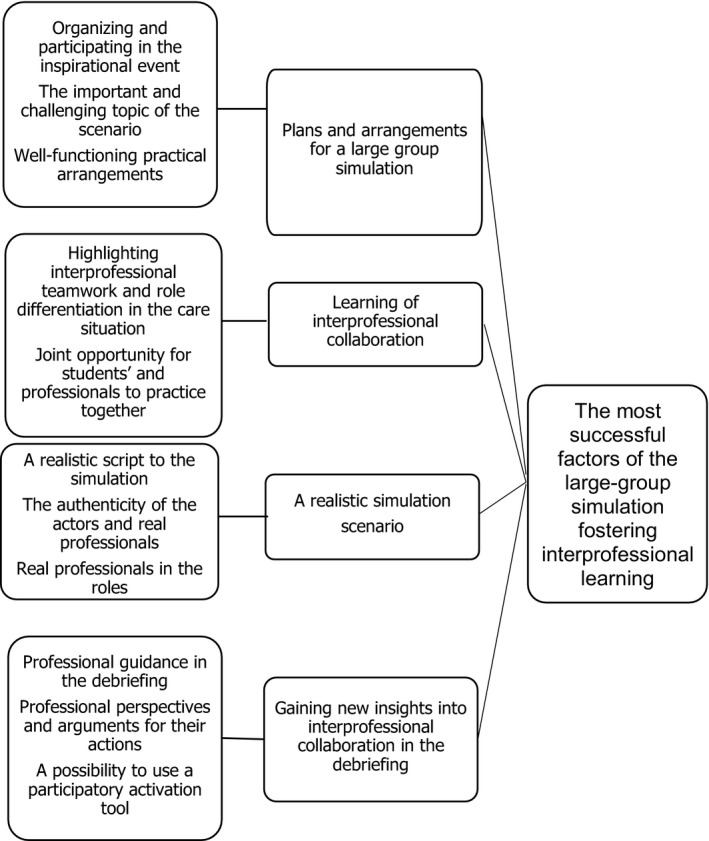
Large‐group simulation fostering interprofessional learning

The analysis of the open‐ended question resulted in forming the main category of *the most successful factors of the large group simulation fostering interprofessional learning* (Figure 1). The main category comprised the following categories: *plans and arrangements for large simulation, learning of interprofessional collaboration, realistic simulation scenario* and *gaining new insight into interprofessional collaboration in the debriefing*.

The participants described the large group simulation as a positive experience of a learning session that was well‐planned and arranged. Furthermore, they were satisfied with the opportunity to attend such an interesting and inspiring learning opportunity. SIDS was an important topic for the scenario and suited the common interprofessional learning objective well. According to the participants, SIDS is a very emotional and sensitive topic, which also makes encountering it challenging, as it is a difficult issue for all the professional groups in health and social care. As this is a challenging situation that no professional ever gets used to, it needs to be practised before facing it in a real‐life context. The participants in the large group simulation felt that the practical arrangements for the simulation were good; the technique worked smoothly, the scheduling was accurate and the simulation proceeded logically. Overall, the event structure was clear and the information given before and during the simulation was sufficient:

A lively, memorable & tangible way of learning, which the university does not offer often enough.

The whole session worked well and was interesting.

The learning of interprofessional collaboration was important, because it highlighted the meaning of multidisciplinarity and interprofessional collaboration in a practical and realistic manner. The actions of several representatives of different professional groups, whose job descriptions and cooperation defined the progress of an interprofessional simulation, fostered the learning experience. The students and professionals also appreciated the opportunity to participate in joint exercises and sharing their opinions:

Functional interprofessionalism, which brought additional perspectives from other professions.

The participants in the large group simulation felt that the simulation scenario was realistic and the incident appealed to the learners in many ways. The acting in the simulation by both the professional actors as the parents and professionals from different fields was considered natural. The fact that the interprofessional team members involved in the simulation were real professionals playing their own professional roles was also appreciated, because it made it easier for the participants to identify with different roles and duties in a stressful situation involving SIDS:

The simulation was so natural and realistic!

The actors and professionals were really good at their roles.

I am certainly much more able to work in such situations after this simulation.

Participants in the large group simulation gained new perspectives and insights and also increased their understanding of interprofessional collaboration. The debriefing was professional and therefore versatile and profound, allowing the participants to focus on broadening their knowledge, thoughts and attitudes related to interprofessional collaboration and different professionals’ work. Additionally, the participants were satisfied with being able to hear the different professionals’ points of view and reasoning concerning the right way to fulfil the demands set by their professions. Moreover, the participants appreciated the opportunity they were given to participate in the debriefing discussion using a participatory activation tool (a mobile application):

You were able to learn the perspectives of many different working areas and professions.

The debriefing was versatile and dealt extensively with the roles of many professional groups.

The professionals were really able to open up their own professional points of view.

You could participate interactively on your mobile phone.

## 5 DISCUSSION

### 5.1 Participants’ experiences and factors promoting learning in the large‐group simulation

As no previous studies are available of simulations carried out with a group as large as the one in the present study, a question on the content of the simulation learning process in a large group emerged in this study. In a study by Reime et al. (2017), the learning processes of those observing a simulation and the persons actively involved in it were compared in an interprofessional small group simulation. According to that study, simulation was also a suitable learning method for observers. Observing the work of other professionals, including the ways they apply their knowledge and skills and engage in interprofessional collaboration, has been found to promote learning, as guided observation is prone to result in personal reflection. Observation is a particularly effective learning method if tasks are set for the observation and planned in accordance with the objectives of the simulation and are subsequently solved by the students during the simulation (Nyström et al., 2016). As learning also occurs through observing the actions of others, this should be accounted for when developing methods for applying simulation pedagogy in large groups. Nevertheless, more research is still needed on how the students observing a simulation participate in the simulation practice and subsequent learning discussion (Husebø, Dieckmann, Rystedt, Soreide, & Friberg, 2013). As all the participants of the present study participated in the simulation as observers, this study provides new insight into learning and teaching in a simulation.

In the present study, the participants felt that their knowledge of and interest in simulation pedagogy increased and they were generally satisfied with the simulation as a learning experience. The participants highlighted many factors considered relevant to their learning, including satisfaction with the implementation and the authenticity of the simulation and debriefing, which promoted forming an understanding of interdisciplinary work. These findings are similar with the results of earlier studies on small‐group simulations, where the participants have found simulation as a motivating learning method (e.g. Costello et al., 2018; Ivey et al., 2018; Roberts & Goodhand, 2018; Zamjahn et al., 2018). The participants of this large‐group simulation appreciated the interprofessional execution of the event, as well as the innovative way of making the simulation available for both students and professionals. Moreover, the participants were inspired by the possibility to learn from one another (Joyal, Katz, Harder, & Dean, 2015; Karpa et al., 2020) and the interaction between the different professions (Wietholter et al., 2017). Practicing interprofessional work enables students to apply learning contents and collaborate with different professions similarly as in working life (Johnston et al., 2017). This study demonstrates that the large group simulation serves as a successful link between education and the working life, therefore, producing a considerable learning effect (cf. Reime et al., 2017).

The above points indicate that there is need for the development of a learning and teaching method involving several target groups in the same simulation. The large group simulation was highly authentic and most of the participants believed that the content of the simulation will be useful for them in their future work. Even though earlier studies on interprofessional simulation have shown similar results (Hovland et al., 2018; Scherer, Myers, O’Connor, & Haskins, 2013), they have been conducted with a limited number of students or professionals (e.g. Saylor, Vernoony, Selekman, & Cowperthwait, 2016). Having real professionals play roles in this large‐group simulation was a unique and successful choice for increasing the reality of the simulation and emphasizing the differences between the professional roles in the scenario.

The participants of this study were satisfied with the comprehensive and professionally supervised debriefing. Debriefing enables linking the experiential and reflective learning that occur in simulation learning. A debriefing session serves as a safe atmosphere that helps students identify what could have been done differently and how the content learned in the simulation can be used in the future (Silén‐Lipponen et al., 2019). Indeed, simulation pedagogy identifies debriefing as a crucial part of the simulation (Dieckmann, Lippert, & Østergaard, 2013) as it allows guiding the participants to learn through reflecting on their own and shared experiences and understanding within a group. In our study, the online‐based system for communication enabled the participation of the audience, that is observers, in the debriefing with their mobile devices. Both students and professionals found this way of participation rewarding. The rationales and perspectives of the different professionals were well‐informed and provided an increased understanding of how different professionals work and approach a crisis. This sort of reflection of experiences in an interprofessional debriefing has also been reported to work well in a previous study (Popkess et al., 2017).

Based on earlier studies, interprofessional learning and teaching as well as interactions between different disciplines are crucial for the development of interprofessional collaboration (Cahill et al., 2013; Tuomela, Heikkilä, Haapanen, Kortekangas‐Savolainen, & Salminen, 2017; WHO, 2010). Teaching cooperation has been found to develop mutual respect between different professional groups and forming an understanding of the stages of the shared care process, clarify the division of tasks and promote communication skills (Fox et al., 2018). Students also seek more interprofessional approaches for learning and practicing their skills and large‐group simulations provide one method for meeting this demand.

However, in this study, differences between disciplines and professions were not explored due to the versatile backgrounds of participants. This requires the development of a questionnaire on the topic. However, it should be noted that, in this study, the participants’ measured background variables (e.g. student or professional) did not correlate with their experiences of the large group simulation even though earlier studies have identified differences in the experiences of various professionals or student groups (Ivey et al., 2018; Saylor et al., 2016). This strongly suggests that this type of a large group simulation can be used simultaneously in both basic education for social and healthcare students as well as in further training provided to different professionals.

### 5.2 Validity and limitations

The data collection of this study was based on a previously used questionnaire, which was modified by the research group for the purposes of this study. Open‐ended questions were used to complement structured, quantitative questions. In addition, direct quotes from the open questions were presented in this report in an aim to ensure the validity of the interpretations made based on them. The strength of the present study is increased using quantitative and qualitative data, that is data triangulation (Polit & Beck, 2018).

This study is limited by the fact that it was based on data concerning only one large group simulation and the participants were not asked about the factors restricting their learning is this sort of a large group simulation. The results can, nonetheless, be used in the development of large‐group simulation training.

## 6 CONCLUSIONS

Social and healthcare services have an increasing demand for an ability of professionals to work jointly across professional boundaries. Sufficient understanding of the role of other professions can positively affect interprofessional collaboration. Many studies have shown that interprofessional learning improves students’ teamwork competence. In this study, new knowledge was obtained about the experiences of professionals and students participating in an interprofessional large‐group simulation. The results showed that the experience was very positive. The results of this study suggest that a large group interprofessional simulation is a valid teaching and learning method for purposes such as acquiring skills related to collaboration or interaction with a client. In addition, all the participants of the present study took part in a simulation as observers and this study therefore provides new, positive insight into learning in a simulation.

## 7 SUGGESTIONS

This study is based on only one large‐group simulation, and no earlier knowledge is available on simulations with comparably high numbers of participants. Therefore, there is need for more research on the large group simulation as a learning method and its effects on the participants’ learning results.

The participants learned and reflected on their learning as observers. This process was supported by an online‐based tool. In the future, more research is needed on how the different roles of simulation learning, including both active involvement in the simulation and observing it, affect the participants’ learning. In addition, further research is needed to determine whether some occupational groups differ in terms of their ability to collaborate with others and what this will require from developing simulation education in the future.

## CONFLICT OF INTEREST

The authors report no conflicts of interest. The authors alone are responsible for the content and writing of this article.

## AUTHOR CONTRIBUTIONS

The authors (Saaranen T, Silén‐Lipponen M, Mönkkönen K and Tiihonen M) have contributed to the design of the study and/or acquisition of data and all authors (Saaranen T, Silén‐Lipponen M, Palkolahti M, Mönkkönen K, Tiihonen M and Sormunen M) have contributed to the analysis and interpretation of data, have drafted the article, read the final version and approve of its publication.
